# Short-Range Guiding Can Result in the Formation of Circular Aggregates in Myxobacteria Populations

**DOI:** 10.1371/journal.pcbi.1004213

**Published:** 2015-04-30

**Authors:** Albertas Janulevicius, Mark van Loosdrecht, Cristian Picioreanu

**Affiliations:** 1 Department of Biotechnology, Delft University of Technology, Delft, The Netherlands; Rice University, UNITED STATES

## Abstract

Myxobacteria are social bacteria that upon starvation form multicellular fruiting bodies whose shape in different species can range from simple mounds to elaborate tree-like structures. The formation of fruiting bodies is a result of collective cell movement on a solid surface. In the course of development, groups of flexible rod-shaped cells form streams and move in circular or spiral patterns to form aggregation centers that can become sites of fruiting body formation. The mechanisms of such cell movement patterns are not well understood. It has been suggested that myxobacterial development depends on short-range contact-mediated interactions between individual cells, i.e. cell aggregation does not require long-range signaling in the population. In this study, by means of a computational mass-spring model, we investigate what types of short-range interactions between cells can result in the formation of streams and circular aggregates during myxobacterial development. We consider short-range head-to-tail guiding between individual cells, whereby movement direction of the head of one cell is affected by the nearby presence of the tail of another cell. We demonstrate that stable streams and circular aggregates can arise only when the trailing cell, in addition to being steered by the tail of the leading cell, is able to speed up to catch up with it. It is suggested that necessary head-to-tail interactions between cells can arise from physical adhesion, response to a diffusible substance or slime extruded by cells, or pulling by motility engine pili. Finally, we consider a case of long-range guiding between cells and show that circular aggregates are able to form without cells increasing speed. These findings present a possibility to discriminate between short-range and long-range guiding mechanisms in myxobacteria by experimentally measuring distribution of cell speeds in circular aggregates.

## Author Summary

Myxobacteria are social bacteria that upon starvation form multicellular fruiting bodies whose shape in different species can range from simple mounds to elaborate tree-like structures. The formation of fruiting bodies is a result of collective cell movement on a solid surface. Since collective cell motility during biological morphogenesis is also common in higher organisms, myxobacteria serve as a relatively simple model organism to study multicellular movement, organization and development. In the course of myxobacterial development, groups of flexible rod-shaped cells form streams and move in circular or spiral patterns to form aggregation centers that can become sites of fruiting body formation. The mechanisms of such cell movement patterns are not well understood. In this study, by means of a computational mass-spring model, we demonstrate that the formation of streams and circular aggregates during myxobacterial development can be explained by short-range head-to-tail guiding between individual cells, whereby movement direction of the head of one cell is affected by the nearby presence of the tail of another cell. We suggest that such interactions between cells can result from physical adhesion, response to a diffusible substance or slime extruded by cells, or the action of cell motility engine.

## Introduction

Myxobacteria are social bacteria that upon starvation form multicellular fruiting bodies whose shape in different species can range from simple mounds to elaborate tree-like structures consisting of 10^5^ − 10^6^ cells [1, 2]. The development of fruiting bodies is a result of collective movement of flexible rod-shaped cells in close contact with one another on a solid surface. After the movement of cells within the fruiting body has stopped, cells differentiate into dessication-resistant spores. Since collective cell motility during morphogenesis is also common in higher organisms [3], myxobacteria serve as a relatively simple model organism to study multicellular movement, organization and development.

In the course of development of myxobacteria, groups of cells move in circular or spiral patterns to form aggregation centers that can become sites of fruiting body formation [4, 5]. Such cell aggregates are dynamic, i.e. they can disperse, split, merge with other aggregates, or stabilize and form a fruiting body [6]. Nascent cell aggregates grow as new cells enter in multicellular streams, where cells are aligned and move in concert [4, 5, 7]. Remarkably, circular and spiral patterns of cell movement are conspicuous during different stages of myxobacterial morphogenesis and can be observed on different spatial scales from several cells to large streams [8–16]. Several adjacent streams can move circularly within the fruiting body in opposite directions [17]. Spores in the fruiting body of *Myxococcus xanthus*, the most studied myxobacterium, have been shown to be organized in spiral patterns, presumably as a result of such movements [5].

The mechanisms of formation of streams and circular or spiral aggregates are not well understood. Circular aggregates can form by a stream of cells trapping itself [8]. Cells have been observed to travel long distances in streams and enter distant aggregates rather that the ones nearby, suggesting that aggregation is not caused by a long-range diffusible signal emitted from aggregation centers [18]. Further, it has been shown that myxobacteria development is regulated by the C-signal that is passed from cell to cell through end-to-end contact [19]. These findings resulted in a hypothesis that myxobacteria aggregation and development depends on short-range contact-mediated communication between cells, i.e. cell aggregation does not require long-range signaling in the population. Recent studies on aggregate merging and dispersal dynamics further argues against the presence of long-range diffusible molecules to signal the aggregation process [6].

Vegetative cells in swarms reverse their direction of gliding by switching leading and trailing poles approximately once every 10 min [20, 21]. In the course of development, due to C-signaling, reversal frequency of cells is reduced and gliding speed is increased. Therefore, cell movement becomes essentially unidirectional at the final stages of development [17, 22, 23]. Søgaard-Andersen and Kaiser [24] proposed that streams form when reversal frequency of cells is decreased due to C-signaling as they come into end-to-end contact. As a result, collective cell movement in roughly the same direction becomes favored. However, this model does not explain what keeps cells in the chain. Moving cell masses and streams can turn and swirl [8], but cells appear to follow one another over long distances and not escape the stream due to random fluctuations in cell orientation [25] or contacts with surrounding cells. A guiding mechanism seems to be present for streams to be stable, i.e. for cells to continue following one another and move as one unit. One possible mechanism of such stability could be a long-range guiding system other than a signal diffusing from aggregation centers. For example, at low cell population densities, cells are often observed to follow slime trails laid down by other cells [26]. This could establish a long-range order required to guide cells into aggregation centers. However, whether and how slime trails could persist in a high-density population, which is the usual state of myxobacteria communities, let alone in three-dimensions, is not clear. Alternatively, cells could employ a short-range guiding mechanism whereby guiding forces act only when cells are in contact or very close to one another. Possible hypothetical mechanisms for short-range guiding could include following slime immediately extruded by another cell, response to a diffusible signal from another cell, physical adhesion between cells or attachment with type IV pili [27].

A number of modeling studies investigated myxobacteria motility (e.g., see [28–30]). However, few of them examined mechanisms of circular motility patterns. Lattice cell simulations showed that streams and ring-shaped aggregates, where cells move in circular tracks, could form as a result of local, short-range contact mediated interactions between cells, whereby rod shaped cells would preferentially turn towards maximizing end-to-end contacts [31–33]. However, modeling approach used there is not mechanically accurate, as cells are perfectly rigid (i.e. cannot bend), can overlap in space and move only in limited number of directions. In this study, by means of a more mechanically accurate two-dimensional (2D) computational mass-spring model developed earlier [34], we investigate how different types of short-range guiding interactions between the leading pole of one bacterium and the trailing pole of another bacterium could affect the formation of patterns in myxobacteria population. In addition, we consider a case of long-range guiding between cells analogous to slime-trail following and compare the resulting patterns with the ones of short-range guided populations.

## Model

**Fig 1:**
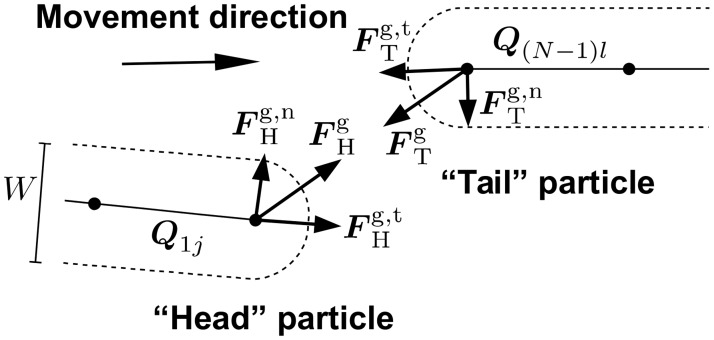
Short-range guiding forces between two cells in the model. Only the leading pole (“head”) of one cell (left) and the trailing pole (“tail”) of another cell (right) are shown. For clarity, the distance between the head and the tail of interacting bacteria is exaggerated. Numbering of line segments ***Q*** that connect adjacent particles on the same bacterium is shown for the case of engine direction *k* ^e^ = 1 (see text and [34] for notation and a detailed explanation of collision resolution algorithm). *W* is cell width, and *d* is the distance between head and tail particles of interacting bacteria.

To model guiding interactions between cells, we use a 2D mass-spring model previously described in [34] with changes to collision response algorithm presented in S1 Text. In brief, a rod-shaped cell is modeled as an array of particles connected by linear and angular springs. Linear springs maintain the distance between particles, and thus the length of a bacterium, whereas angular springs (characterized by angular spring constant *k* ^a^) resist bending of a cell. Cells glide on a substratum powered by engine forces and change their direction of movement as a result of collisions with other cells. Here, only the distributed engine is considered (i.e. engine with forces distributed along the whole length of a cell), given recent evidence strongly supporting its existence [35–37]. In addition to the features described in the basic model, here we introduce and study three kinds of short-range guiding forces (Fig 1). First, *adhesion* between the leading pole (“head”) of one cell and the trailing pole (“tail”) of another cell is considered. Thereby, adhesion forces between a pair of line segments that connects particles in bacteria are introduced only when the head of one bacterium and the tail of another (or the same) bacterium are involved. If both interacting cells have polarity *k* ^e^ = 1, the head of bacterium *j* is the point *P*_1_ = 0 on segment ***Q***_1*j*_, and the tail of bacterium *l* is the point *P*_2_ = 1 on segment ***Q***_(*N* − 1)*l*_ (see [34] for notation). Thus, when the smallest distance between the two segments *d* is *W* < *d* < *d*_g_, where *d*_g_ is maximum guiding (in this case, adhesion) distance and *W* is cell width, adhesion forces to respective head and tail particles of interacting bacteria are introduced (Fig 1, forces marked $F_{\text{H}}^{\text{g}}$ and $F_{\text{T}}^{\text{g}}$). The adhesion forces are described by the same 4 equations that govern collision response [34], with *k* ^c^ replaced by $k^{\text{g}}=F_{\text{max}}^{\text{g}}/d_{\text{g}}$, where $F_{\text{max}}^{\text{g}}$ is the maximum magnitude of the guiding force (exerted when two segments are separated by distance *d*_g_). Essentially, adhesion in the model is collision response working in reverse, i.e. attracting cells when *d* becomes larger than *W*. As a result of these forces, the head of the trailing cell will tend to turn towards the tail of the leading cell when the distance between them is small enough, due to the normal component of adhesion force on the head particle ($F_{\text{H}}^{\text{g,n}}$ in Fig 1). In addition, the component of adhesion force along the tangent of trailing bacterium body ($F_{\text{H}}^{\text{g,t}}$ in Fig 1) will result in increased speed of the trailing bacterium (i.e. the leading cell will pull the trailing cell forward). As adhesion forces work in action-reaction pairs, the tail of the leading cell will also turn towards the head of the trailing cell and the speed of the leading cell will tend to decrease (due to normal and tangent component of adhesion force respectively, $F_{\text{T}}^{\text{g,n}}$ and $F_{\text{T}}^{\text{g,t}}$ in Fig 1). Overall, when *d* < *W* (i.e. when cells overlap), only collision forces would act to separate the cells, and there will be no adhesion forces. Collision and adhesion forces would be zero when the head of the trailing cell touches the tail of the leading cell (i.e. when *d* = *W*). As distance between head and tail particles *d* increases beyond *W*, the magnitude of adhesion forces increases linearly until distance *d*_g_, where the magnitude of adhesion forces gets its maximal value $F_{\text{max}}^{\text{g}}$. Beyond *d*_g_, adhesion forces would be zero and thus cells will have no guiding interactions.

A second type of short-range guiding force represents *active following*, whereby the force described for *adhesion* is added only to the head of the trailing cell, but not to the tail of the leading cell (i.e. only $F_{\text{H}}^{\text{g}}$ in Fig 1 is added). It models the effect of the trailing cell responding to the presence of the tail of the leading cell by actively moving in its direction, but having no effect on the movement of the leading cell.

A third type of short-range guiding force is *passive following (steering)*, whereby the force to the head particle of the trailing cell is added only in the direction normal to bacteria body $\hat{n}$ (${\text{F}}_{\text{H}}^{\text{g,n}}$ in Fig 1). By this, only the steering effect on the head of the trailing cell is modeled, i.e. turning the tip of the cell left or right with respect to the normal trajectory of the cell, but having no effect on cell speed.

In addition, we also consider a case of long-range guiding that is analogous to slime trail following by myxobacteria cells. To model slime trails, a square grid with elements of side Δ*x* is defined on the substratum. Each grid element can contain a unit vector ***s*** indicating slime trail direction at that location, or a zero vector, if no slime trail is present [38]. When the head particle of a bacterium glides over a grid element containing a slime trail ***s***, the guiding force on the head particle is introduced to reorient the leading tip of that cell along the slime trail. Since a cell can glide in both directions along the slime trail, the guiding force is defined to turn the cell by an acute angle [26]. Thus, if the orientation of the leading tip ***o*** is defined as the tangent to bacterial body at the leading particle (i.e. ${\hat{t}}_{1}$ when engine direction *k* ^e^ = 1 and ${\hat{t}}_{N}$ when *k* ^e^ = −1) and $F_{\text{max}}^{\text{s}}$ is the maximal magnitude of guiding force, a guiding force $\text{sgn}(\text{o}\cdot \text{s})F_{\text{max}}^{\text{s}}\text{s}$ is found and its component in the direction of $\hat{\text{n}}$ is added to the leading particle. The applied force is similar to passive following described above, because the force only orients the tip of the cell along the slime trail, without affecting cell speed along tangent $\hat{\text{t}}$. After each integration step, the deposition of slime by the rear of the bacterium is modeled by assigning a tangent $\hat{\text{t}}$ at the rear particle to slime trail ***s*** at a grid element below. The deposition of slime overrides the previous value of slime trail direction at that grid location. Slime trails at each grid location persist until overridden by other cells.

The parameters used in the simulations are the same as in [34], with the addition of extra parameters describing guiding forces. The value $F_{\text{max}}^{\text{g}}$ was chosen to be 200 pN, unless stated otherwise, and *d*_g_ = 0.25 μm (i.e. half of bacterium width *W*). Since guiding forces not only steer the head of the cell, but can also speed up the cell, the value of $F_{\text{max}}^{\text{g}}$ was chosen in such a way that the speed-up due to the guiding force would be roughly within experimentally observed speed increase of myxobacteria cells during development, 1.5–2.5 times [23]. $F_{\text{max}}^{\text{g}}=200\;\text{pN}$ results in 3-fold maximum increase of speed (engine force of 100 pN and maximum guiding force of 200 pN results in maximal 3*v*_b_ speed). For long-range guiding simulations, $F_{\text{max}}^{\text{s}}$ was also set to 200 pN and Δ*x* = 0.25 μm (half of bacterium width *W*). Since bending stiffness of myxobacteria cells have not been experimentally determined, but only theoretically estimated for *M. xanthus* [34, 39], a wide range of angular spring stiffness values *k* ^a^ were studied in the simulations: 1 × 10^−18^, 1 × 10^−17^, 1 × 10^−16^ and 1 × 10^−15^ N·m. They correspond to cell bending stiffness (*B*) values of 7 × 10^−25^ J·m (referred to in the text as “very flexible”), 6 × 10^−24^ J·m (“flexible”), 6 × 10^−23^ J·m (“rigid”) and 6 × 10^−22^ J·m (“very rigid”), respectively. Further, both low density 5 × 10^6^ cells/cm^2^ and high-density 4 × 10^7^ cells/cm^2^ populations were studied. For a low density population simulation, the collision stiffness between cells was set as in [34], *k* ^c^ = 0.01 N·m^−1^. For high density populations, the collision stiffness had to be reduced to *k* ^c^ = 0.002 N·m^−1^, because high collision stiffness blocks the movement of cells in a crowded environment.

To analyze cell movement, cell speeds and strain energies due to collisions between cells were shown for every line segment in the bacterium. Speed of a line segment was defined as an average speed of two particles at the ends of the segment. To find strain energies, for every two segments that overlap due to collision (i.e. the when the smallest distance between segments *d* < *W*), potential energy of the collision response spring (1/2)*k* ^c^(*d* − *W*)^2^ was calculated and one half of the value was added to both segments involved.

## Results

This study shows that both short-range and long-range guiding between cells have a marked effect on the patterns observed in the model myxobacteria population.

### Short-range guiding

**Fig 2:**
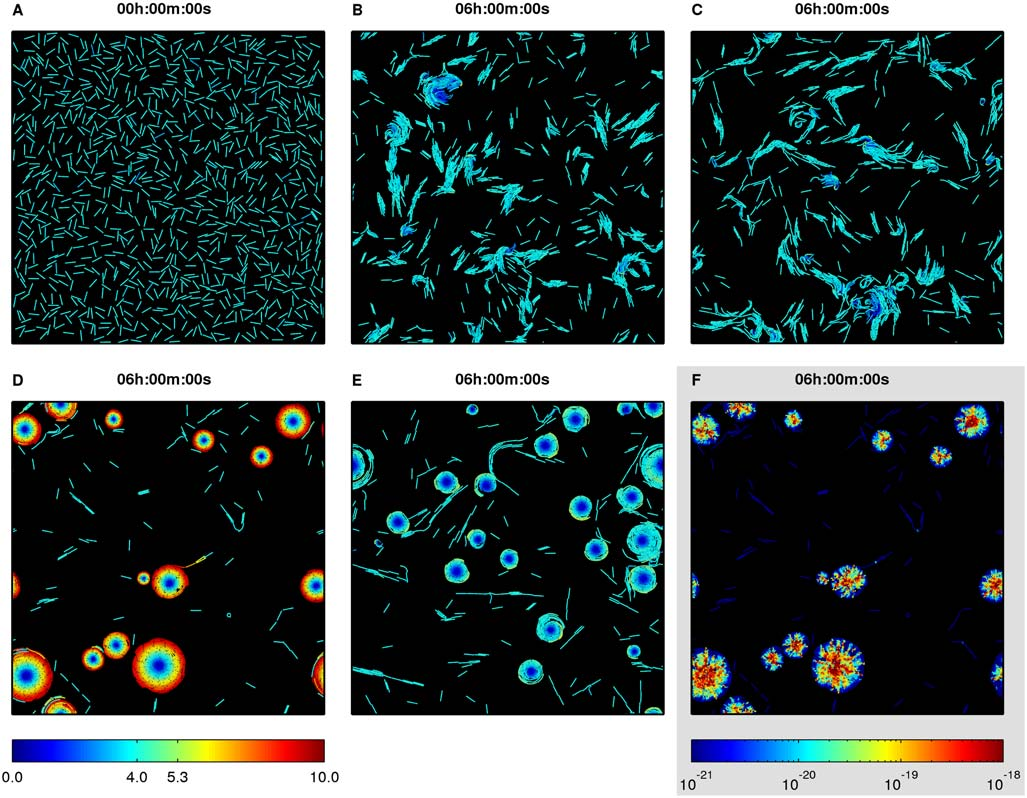
Effect of short-range guiding forces on cell movement patterns in a low-density population of flexible cells. (A) Initial random cell configuration. (B-E) Final configuration of a population at 6 h (B) non-guided cells, (C) cells with passive following, (D) cells with active following, (E) cells with head-to-tail adhesion. In (A-E) color indicates speed of individual cells (colorbar at the bottom left, μm·min^−1^). (F) Strain energies due to cell overlap in circular aggregates of cells with active following (colorbar at the bottom right, J).

Firstly, the effect of different types of short-range guiding forces on cell movement patterns of a low-density population of non-reversing cells was studied. All cells were initially placed on a planar substratum with random positions and orientations (Fig 2A), and cell movement was simulated for 6 hours. A population of flexible non-guided cells at 6 hours formed clusters (Fig 2B and S1 Video), whereas the presence of steering forces between cells (passive following) resulted in occasional chains of cells between clusters and small unstable circular structures that quickly dissipated (Fig 2C and S2 Video). However, cells with active following and head-to-tail adhesion formed stable rotating circular aggregates (Fig 2D and S3 Video, and Fig 2E and S4 Video, respectively). During the process of aggregate formation, streams of cells were first formed from randomly distributed cells. A stream could collide with other streams, turn, move in circular trajectories, close in upon itself and trap the leading cells. The rest of stream cells then swirled around the trapped cells. The seed of rotation could also be formed by several cells swirling around a fixed point. Later, additional cells or entire streams could join in to increase the size of the aggregate. Within the aggregate, cells were arranged spirally and new streams joined in by following the freely exposed tail of a cell at the aggregate edge. The decrease of guiding force $F_{\text{max}}^{\text{g}}$ from 200 pN to 100 pN (referred to as weak guiding) resulted in a more dynamic population that was less likely to form stable rotating aggregates. In such a population circular aggregates were smaller, could dissipate, and streams could leave one aggregate and join another (S5 Video). Interestingly, when a cell at the edge of the aggregate left, it often had a chain of trailing cells behind it.

#### Effect of cell bending stiffness

Non-guided cell populations form clusters whose sizes appear to increase as cell bending stiffness increases (S2 Fig), i.e. stiffer cells result in larger, more stable clusters. Similarly, the size of circular aggregates of cells with active following appears to increase as cell bending stiffness increases, and as a consequence, the number of aggregates in the computational domain decreases (S3 Fig). Very flexible cells (S3A Fig, S6 Video) form many small and stable circular aggregates due to their tendency to easier form a seed for a rotating aggregate. The size of these aggregates cannot increase due to unavailability of free cells to join, because most of the cells are trapped in stable small aggregates. Conversely, very stiff cells do not form circular aggregates (S3D Fig and S7 Video) because rigid cells cannot bend and are unlikely to generate a rotation seed or initiate the rotation of the entire stream.

#### Circular aggregates as rigid bodies

Stable rotating aggregates appear to rotate as rigid bodies, i.e. cells do not slide laterally within the aggregate and cells in the center move slower than cells at the edge of the aggregate (Fig 2D and 2E, S3A–S3C Fig and S3 Video, S4 Video and S5 Video). Furthermore, speed of cells at the aggregate edge is larger than the speed of a freely moving bacterium, *v*_b_, and appears not to depend on the aggregate size. As a consequence, because the speed of points at the edge of a rotating rigid body must be *v*(*R*) = *ωR*, where *R* is the distance of the point from the rotation axis and *ω* is angular speed of the rotating body, larger aggregates rotate with smaller angular speed, and angular speed of a growing aggregate decreases. To validate this result, we formulated a continuous rigid body model of a rotating myxobacteria aggregate (S2 Text). The rigid body model predicts that speed of cells at the aggregate edge in the head-to-tail adhesion case should be *ωR* = *v*(*R*) = (4/3)*v*_b_ = 5.3 μm·min^−1^, whereas in the active following case (given $F_{\text{max}}^{\text{g}}=200\;\text{pN}$) maximum speed should be *ωR* = *v*(*R*) = 4*v*_b_ = 16 μm·min^−1^, well in agreement with the simulation results (Fig 2D and 2E and S3 Video and S4 Video respectively).

The finding that cells at the edge of a nascent rotating aggregate move faster than their equilibrium speed can explain why large stable rotating aggregates do not form with only steering forces present. Since guiding forces in the model are short-range, a trailing cell must continuously be within a short distance from the tail of a leading cell to be steered to follow it. If a cell happens to come close to the freely exposed tail of another cell at the aggregate edge, it must speed-up to remain within a short distance from that cell to be steered to move in a circular path and become a part of the aggregate. Otherwise, if the incoming cell is unable to catch up with the faster-moving cell at the aggregate edge, the distance between them increases until guiding interaction is lost. Steering forces can only turn the head of the cell in the direction normal to the bacterium body, but cannot act to increase cell speed. Conversely, head-to-tail adhesion and active following forces do speed up trailing cells.

Finally, the increase in cell speed beyond equilibrium speed at the edges of circular aggregates can be explained by spiral arrangement of cells inside the aggregates. In a spiral arrangement, each trailing cell is located at a slightly larger distance from the rotation axis than the respective leading cell (S5 Fig). Each cell, however, moves circularly around the rotation axis. Therefore, a trailing cell must travel a longer distance than the leading cell in order to maintain a small separation distance necessary for short-range guiding forces to act and thus for the aggregate to be stable. As a result, in a spiral chain of cells, each cell must move faster than the one in front of it. As a cell in the model can only speed-up due to guiding forces, cell speeds in the aggregate can increase only to the level that guiding forces can sustain.

#### Stress inside circular aggregates

Overlaps between cells tend to be higher towards the center of rotating aggregates, which implies larger stresses that cells undergo due to being squeezed by surrounding cells. Fig 2F and S8 Video show strain energies within the cells due to overlap between different bacteria (i.e. energies stored in the collision response springs). Whereas during cluster formation in non-guided populations of cells strain energies during the collision can reach temporary high values, inside circular aggregates of guided cells high stresses are constant. This is consistent with the fact that in the core of the aggregate, cells move slower than their equilibrium speed, and thus the engine force of a cell is counteracted by pushing force from surrounding cells.

#### Robustness of circular aggregates

An interesting question is how robust circular aggregates are with respect to their size and whether stable aggregates can form in populations of high density. We performed simulations where initially all cells in a population were arranged spirally to form a circular aggregate (S6A Fig). Interestingly, prearranged aggregates of rigid and very rigid cells were relatively stable, although they did lose some cells (S6B Fig and S9 Video). However, the aggregates of the same initial size made of flexible and very flexible cells split after some time into several smaller aggregates and swirling streams (hollow aggregates) (S6C Fig and S10 Video). It appears that flexible cells under high stresses inside the aggregate can bend enough to start forming separate rotation seeds. This suggests the existence of a different optimum aggregate size for cells of different flexibility.

**Fig 3:**
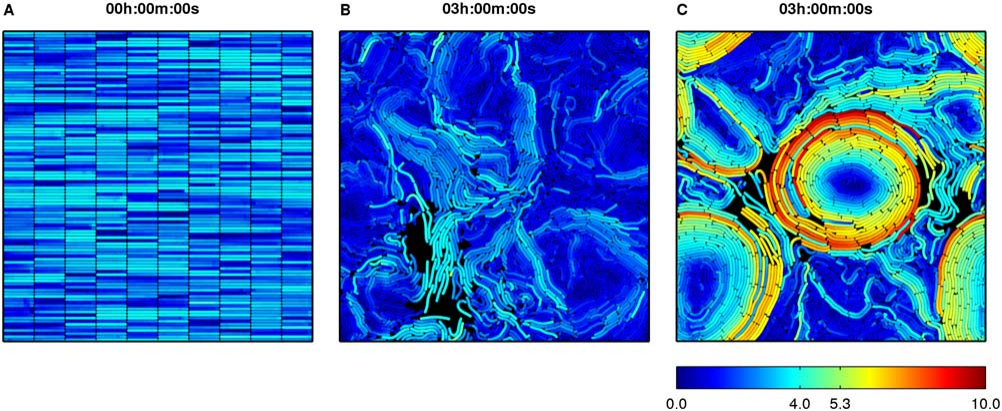
Effect of short-range guiding forces on cell movement patterns in a high-density population of flexible cells. (A) Initial configuration. Cells are aligned, but oriented randomly. (B). Final configuration of a population of non-guided cells at 3 h. (C). Final configuration of a population of cells with active following at 3 h. Color indicates speed of individual cells (colorbar at the bottom, μm·min^−1^).

It was further explored whether the formation of circular aggregates was robust in high density populations. All cells were initially densely packed, aligned, but with random orientations (Fig 3A). A population of non-guided cells did not show any discernible characteristic movement pattern for all bending stiffness values except very rigid ones (Fig 3B, S11 Video). Interestingly, very rigid cells were able to sort themselves into straight streams (S12 Video). However, in populations of flexible and very flexible cells with head-to-tail adhesion or active following there was a visible stable circular movement within the population (Fig 3C, S13 Video). Rigid and very rigid cells sorted themselves into adjacent straight streams (S14 Video), similar to the streams of rigid non-guided cells. While at low population density the size of circular aggregates increased with cell rigidity, at high density rigid guided cells might not have formed circular aggregates because the domain of the simulation was too small. Interestingly, in populations with active following, whole streams moved with a speed larger than the equilibrium speed *v*_b_, because each cell was responding to the speed up of the cell in front of it by increasing its own speed. Due to the periodic boundary adopted in the model, it resulted in a self-sustained speedup of the whole stream.

#### Effect of cell reversal

Results presented so far have been obtained with populations of non-reversing cells. Reversing cells (reversal period *T*_R_ = 10 min) did not form circular aggregates even with guiding forces present. However, if in addition to guiding forces, cell reversals were suppressed when a head-to-tail interaction between cells took place (i.e. when the distance between head and tail of two bacteria became smaller than *d*_g_), circular aggregates did form (S15 Video).

### Long-range guiding

**Fig 4:**
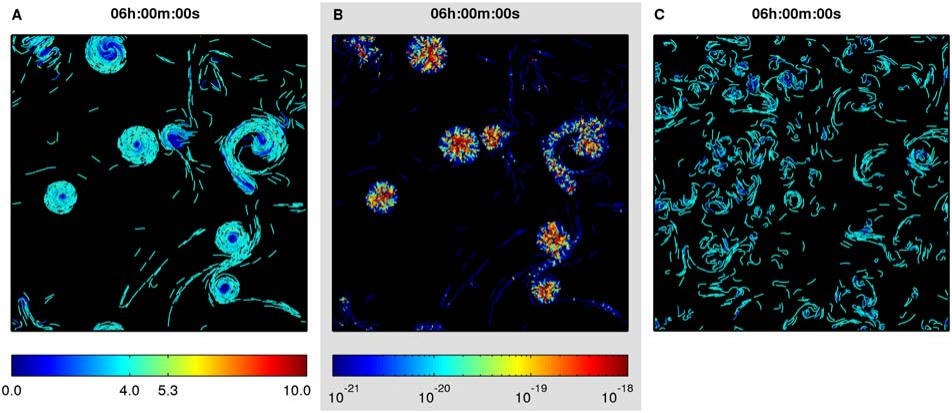
Effect of long-range guiding forces on cell movement patterns in a low-density population. Initial configuration of cells is random, as in Fig 2A. (A) Final configuration of a population of rigid cells at 6 h. Color indicates speed of individual cells, μm·min^−1^. (B) Strain energies due to cell overlap in population of rigid cells, J. (C) Final configuration of a population of flexible cells at 6 h. Color indicates speed of individual cells (see colorbar in (A), μm·min^−1^).

Stable circular aggregates also form in a population of rigid non-reversing cells with long-range guiding (Fig 4A and S16 Video). However, in contrast to short-range guided aggregates, most of the cells traveled with equilibrium speed, including the ones at aggregate edges, as guiding forces affected only cell movement direction but not cell speed. Further, aggregates appear to be less tightly packed, i.e. they contain more voids than short-range guided aggregates. Stress accumulation patterns inside aggregates, however, are similar in both cases (Fig 4B and Fig 2F). Interestingly, flexible cells also exhibited marked circular movement and produced small short-lived circular aggregates, but they were unstable (Fig 4C and S17 Video). The instability could be explained by easier bending of flexible cells under stress inside nascent aggregates. As cells can travel in both directions on a slime trail, bent flexible cells can switch their movement direction to the opposite, squeeze in or be pushed through voids in the aggregate and thus disturb the circular arrangement of slime trails.

## Discussion

The mechanisms of myxobacteria aggregation during fruiting body formation are not well understood. Non-linear patterns of movement of myxobacteria cell masses and streams imply the existence of some sort of guiding mechanisms that keep the cells moving as one unit and direct them into aggregation centers [8]. It is not known whether these guiding mechanisms are long-range or short-range. In this study, by means of a computational mechanical mass-spring model, we demonstrate that short-range guiding between the head and the tail of two myxobacteria cells in close contact are sufficient to produce stable streams and circular or spiral aggregates in model myxobacteria populations.

Many features of cell movement that are present in our short-range guiding simulations are also observed in experimental videos. Multicellular cell masses (streams) in the simulations can travel in straight lines, or, when colliding with other streams or clusters of cells, can wave and swirl (S5 Video, [8, 11, 14]). A circular aggregate in the simulations often forms when a stream turns, closes in upon itself and traps the leading cells, a situation also observed in experimental videos (S3 Video; [8]). Simulated circular aggregates exhibit rotational movements (S3 Video). Similarly, circular and spiral movement is often observed in developing myxobacteria [9, 12]. In fact, fruiting bodies often develop in places where such spiral aggregates initially form [5]. Additional streams of cells join existing aggregates to increase their size (S3 Video, [23]). In the simulations, a circular aggregate sometimes forms from a rotation seed of several flexible cells (S3 Video and S6 Video). Similar small rotating cell clusters have been observed experimentally [10, 15, 40, 41]. Furthermore, a smaller magnitude of guiding forces results in a more dynamic aggregate behavior: simulated streams can travel from one aggregation center to another, aggregates can dissipate, split or join with other aggregates (S5 Video). Our results show that stability of large aggregates increases with increasing cell rigidity, whereas more flexible cells tend to form separate rotation seeds inside aggregates due to easier bending and thus induce splitting of a large aggregate (S9 Video and S10 Video). It has been experimentally observed that the size of initial aggregates of different myxobacteria species differs [42]. Our results suggest that it might be the result of different bending stiffness of cells of different species [43]. Finally, we also observed the formation of hollow aggregates and adjacent streams swirling inside aggregates in opposite directions (S10 Video, [4, 17]). The formation of ring-shaped aggregates, where cells move in circular tracks, both clockwise and counter-clockwise, was also observed in lattice cell modeling studies [31–33]. In these studies cells preferentially turn towards maximizing end-to-end contacts with other cells and therefore the interactions between cells are effectively similar to guiding forces in our model.

Interestingly, short-range guided circular aggregates in our model rotate as rigid bodies, i.e. cells within the aggregate do not slide laterally past one another. As a consequence, the further from the rotation axis cells are located, the faster they travel. Furthermore, cells at the edge of a simulated aggregate and cells in the incoming streams move faster than the equilibrium cell speed (S3 Video). Therefore, in order for stable rotating aggregates to form, short-range guiding must act in such a way that the trailing cell is both turned towards the tail of the leading cell and sped up to catch up with it. Otherwise, because guiding interactions are short-range (half the cell width), the faster moving leading cell will escape the trailing cell and the short-range guiding interaction will be lost. It has been shown that during myxobacterial development average speed of cells does increase due to interaction with other cells [23, 44], but unfortunately it was not reported whether cell speed depended on cell location within circular aggregates or streams.

Long-range guided populations were also able to form circular aggregates in the simulations, but in contrast to short-range guided populations, cell speed increase was not required, as most cells inside aggregates traveled with equilibrium speed, including the ones at aggregate edges (S16 Video). This finding could be used to experimentally discriminate between short-range and long-range guiding mechanisms present in myxobacteria. Observation of currently available experimental videos does not allow to tell conclusively whether circular aggregates rotate fully or partially as rigid bodies and whether cell speed increases with increasing distance from the rotation axis [9, 12]. Experimentally tagging part of the cells in the population with fluorescent markers could be used to obtain such data. Another experimentally testable prediction of the model is that cell speed at the short-range guided aggregate edge is independent of the aggregate size. This means that smaller aggregates rotate with larger angular speeds, and increase of the aggregate size due to incoming streams will result in decrease of the angular speed of aggregate rotation.

To our knowledge, there is no experimental evidence about the existence of short-range guiding interactions between a head and a tail of two myxobacteria cells. The model proposed in this study does not imply any particular short-range guiding mechanism for myxobacteria aggregation, as long as the interaction would both steer the trailing cell and adjust its speed. One possibility could be mechanical adhesion force between a head and a tail of two myxobacteria, or physical link between cells by type IV pili. A gliding *M. xanthus* cell extends type IV pili that originate at the leading pole, attach to neighboring cells and pull to produce motility force [27, 45]. Groups of myxobacteria cells are usually well aligned [46], therefore it is likely that the extended pilus will attach to the rear of the leading cell. Alternatively, the leading pole of a trailing cell could respond to a diffusible substance or slime secreted from the rear of the leading cell. For a short-range interaction, such a substance should diffuse slow enough to form gradients on the spatial scale of cell width and should break down quickly not to interfere with signaling between other cells at the same location at a later time. For example, lipids could satisfy slow diffusion requirement [6]. In such a scenario, a trailing cell would turn and adjust its speed based on the concentration of the diffusing substance. It has been shown that bacteria are not too small for spatial sensing of chemical gradients [47]. Furthermore, it has been observed that in low-density populations myxobacteria cells tend to follow slime trails produced by other myxobacteria [26]. It is not clear, however, whether slime trails could persist for a long time in high-density population, a usual state of myxobacterial communities. At high cell densities, a particular spot on a substratum is continuously overrun by other cells and existing slime trails thus would be overridden. Therefore, it is possible that slime trails in high-density populations are short-lived and extend no longer than the distance between adjacent cells. For short-range guiding, the trailing cell should follow only the new slime immediately secreted by the leading cell, but not the old slime. If slime-contained signaling molecule were broken down quickly after slime extrusion from the cell rear, its concentration would show slime age, and therefore, the distance to the leading cell that produced it. If, further, a trailing cell responded to older slime by increasing speed, the situation would be akin to active following considered in our simulations, as cell speed would be dependent on the distance between interacting cells. Furthermore, type IV pili can also attach to slime left behind by other cells [45]. If the pili attached only to immediately extruded slime and the force of pulling were proportional to the length of extended pilus, it would also present active following. It has also been shown that myxobacteria development depends on contact mediated C-signaling [19]. C-signal is relayed by the end-to-end contact between cells [48], and one of its effects is to decrease cell reversal frequency [23]. C-signal mutants are unable to aggregate, or the aggregates that form quickly dissipate [49]. These results are consistent with C-signal acting as a part of guiding mechanism suggested by our model. In our simulations, weak guiding forces resulted in the formation of very dynamic aggregates that could easily disperse (S5 Video).

Although a real fruiting body develops in three dimensions, at the initial stages of aggregation cells appear to move in independent monolayers that are stacked on top of one another [5, 49]. This observation suggests the presence of forces that keep cells confined to two-dimensional sheets and do not allow them to escape crowded environment by moving upward. It also justifies a 2D model in this study and explains how cell trapping is possible when streams close in upon themselves. Further, our simulations show that mechanical stress accumulates inside circular aggregates because cells are trapped and squeezed. It has been experimentally observed that when a second layer forms on top of the original monolayer of *M. xanthus* cells, cells leave the base layer at one point [49]. Our results suggest that this phenomenon may occur when mechanical stress reaches a critical value at some point inside the aggregate and cells at that point are propelled upwards. Consistent with this idea is the observation that fruiting bodies develop at the places of traffic jams [50] or where spiral aggregates initially form [5].

During vegetative swarm phase of myxobacterial life cycle, cells reverse their direction of gliding by switching the leading and trailing pole approximately once every 10 min [20, 21]. In the course of development, reversal frequency of cells decreases and cell movement become essentially unidirectional at the final stages of development [17, 22, 23]. Reversing cells, in contrast to non-reversing cells, are unable to form circular aggregates in our simulations. This result is in a good agreement with experimental observations that circular aggregates do not form during vegetative stage of myxobacterial life cycle [46], but only during fruiting body development. Furthermore, our study suggests an extension of the conceptual model whereby cell streams form when cell reversal frequency is reduced due to contact-mediated C-signaling as two cells come into end-to-end contact [24]. It was proposed that collective cell movement in roughly the same direction would be favored as a result. However, this model does not address the question of what keeps cells in the chain. Streams can turn and swirl [8], but cells appear to follow one another over long distances and not escape due to contacts with surrounding cells or random fluctuations in cell orientation [25]. Our results show that cells with only suppressed reversals would not be able to form streams from initially randomly distributed reversing cells. However, the presence of guiding interactions in addition to reversal suppression allows for the formation of stable streams and circular aggregates (S15 Video). Interestingly, in our high-density population simulations, initially aligned but randomly oriented rigid cells could mechanically sort into adjacent streams of cells moving in the same direction (S12 Video), but it is not clear whether this effect occured due to a relatively small simulation domain. Furthermore, bending stiffness of myxobacteria cells has not been determined experimentally, but evidence suggests that for *M. xanthus* it is closer to the “flexible” value used in our simulations [51]. Guiding forces allow cells to form stable streams and circular aggregates independently of bending stiffness value and initial cell configuration.

## Supporting Information

Supporting videos are encoded in H.264 format. Please note that not all media players can handle this format by default. Installation of a proper codec is needed in such cases. For the best viewing experience, we recommend VLC Media Player, freely available for a number of different platforms (http://www.videolan.org/vlc/).

S1 Text Modifications of the original collision response algorithm [34]. (PDF)

S2 Text Continuous rigid body model of a circular aggregate. (PDF)

S1 Fig Collision detection and response issues in the original model [34]. (A) Double contact forces arise because two adjacent segments share the same endpoint. (B) Friction forces arise when a segment is within contact distance with two adjacent segments on the same bacterium. *AB* is the shortest distance between segments *a* and *b* (i.e. *A* and *B* are contact points on the respective segments), *AC* is the shortest distance between segments *a* and *c*. $F_{a,AB}^{\text{c}}$ is the collision force that acts on segment *a* due to segment *b*. $F_{a,AC}^{\text{c}}$ is the collision force that acts on segment *a* due to segment *c*.
(TIFF)

S2 Fig Cell movement patterns in a low-density population of non-guided cells for different bending stiffness values. Cells were initially arranged randomly, as in Fig 3A. Final configuration of a population at 6 h is shown. Color indicates speed of individual cells (colorbar at the bottom, μm·min^−1^).
(TIFF)

S3 Fig Cell movement patterns in a low-density population of cells with active following for different bending stiffness values. Cells were initially arranged randomly, as in Fig 3A. Final configuration of a population at 6 h is shown. Color indicates speed of individual cells (colorbar at the bottom, μm·min^−1^).
(TIFF)

S4 Fig Continuous rigid body model of a circular aggregate. A small element of a circular sector (shaded), direction of its velocity (***v***), and directions of engine force (***F*** ^e^) and drag force (***F*** ^d^) that act on the element are shown.
(TIFF)

S5 Fig Distances traveled by leading and trailing cells in a stable rotating circular aggregate. For simplicity, cells are represented as points. In a spiral arrangement of cells, each trailing cell (T) is located slightly further from the rotation axis than the respective leading cell (L). Each cell moves circularly around the rotation axis. The leading and the trailing cells must maintain a small separation for short-range guiding forces to work. Therefore, between time points 1 and 2, when the leading cell travels the distance *rθ*, the trailing cell must travel a longer distance, (*r* + *dr*)*θ*.
(TIFF)

S6 Fig Cell movement patterns in a population of initially spirally arranged cells with active following. (A) Initial cell configuration. (B) Final configuration of rigid cells at 6 h. (C) Final configuration of flexible cells at 6 h. Color indicates speed of individual cells (colorbar at the bottom, μm·min^−1^).
(TIFF)

S1 Video Cell movement patterns in a low-density population of non-guided flexible cells. Color indicates speed of individual cells (colorbar at the bottom, μm·min^−1^).
(AVI)

S2 Video Cell movement patterns in a low-density population of flexible cells with passive following. Color indicates speed of individual cells (colorbar at the bottom, μm·min^−1^).
(AVI)

S3 Video Cell movement patterns in a low-density population of flexible cells with active following. Color indicates speed of individual cells (colorbar at the bottom, μm·min^−1^).
(AVI)

S4 Video Cell movement patterns in a low-density population of flexible cells with head-to-tail adhesion. Color indicates speed of individual cells (colorbar at the bottom, μm·min^−1^).
(AVI)

S5 Video Cell movement patterns in a low-density population of flexible cells with weak active following. Color indicates speed of individual cells (colorbar at the bottom, μm·min^−1^).
(AVI)

S6 Video Cell movement patterns in a low-density population of very flexible cells with active following. Color indicates speed of individual cells (colorbar at the bottom, μm·min^−1^).
(AVI)

S7 Video Cell movement patterns in a low-density population of very rigid cells with active following. Color indicates speed of individual cells (colorbar at the bottom, μm·min^−1^).
(AVI)

S8 Video Strain energy due to cell overlap in a low-density population of flexible cells with active following (colorbar, J). (AVI)

S9 Video Cell movement patterns in a population of rigid, initially spirally arranged cells with active following. Color indicates speed of individual cells (colorbar at the bottom, μm·min^−1^).
(AVI)

S10 Video Cell movement patterns in a population of flexible, initially spirally arranged cells with active following. Color indicates speed of individual cells (colorbar at the bottom, μm·min^−1^).
(AVI)

S11 Video Cell movement patterns in a high-density population of non-guided flexible cells. Color indicates speed of individual cells (colorbar at the bottom, μm·min^−1^).
(AVI)

S12 Video Cell movement patterns in a high-density population of non-guided very rigid cells. Color indicates speed of individual cells (colorbar at the bottom, μm·min^−1^).
(AVI)

S13 Video Cell movement patterns in a high-density population of flexible cells with active following. Color indicates speed of individual cells (colorbar at the bottom, μm·min^−1^).
(AVI)

S14 Video Cell movement patterns in a high-density population of very rigid cells with active following. Color indicates speed of individual cells (colorbar at the bottom, μm·min^−1^).
(AVI)

S15 Video Cell movement patterns in a low-density population of flexible, reversing cells with active following and reversal suppression. Color indicates speed of individual cells (colorbar at the bottom, μm·min^−1^).
(AVI)

S16 Video Cell movement patterns in a low-density population of long-range guided rigid cells. Color indicates speed of individual cells (colorbar at the bottom, μm·min^−1^).
(AVI)

S17 Video Cell movement patterns in a low-density population of long-range guided flexible cells. Color indicates speed of individual cells (colorbar at the bottom, μm·min^−1^).
(AVI)
