# Supplementary material for: Short-Range Guiding Can Result in the Formation of Circular Aggregates in Myxobacteria Populations
Source: PLoS Comput Biol. 2015 Apr 30;11(4):e1004213. doi: 10.1371/journal.pcbi.1004213 (PMC4415783; doi:10.1371/journal.pcbi.1004213)
Supplement: S1 Text — (PDF) [file pcbi.1004213.s001.pdf]

Supporting Text S1

Short-range Guiding can Result in the Formation of Circular Aggregates in Myxobacteria Populations

Albertas Janulevicius, Mark van Loosdrecht, Cristian Picioreanu

Modifications of the original collision response algorithm. The collision response algorithm presented in [1] was modified to avoid undesirable effects arising when contact forces are produced by adjacent segments, i.e. segments sharing the same endpoint. For example, in the situation shown in S1A Fig, contact forces between bacteria would be added twice, because according to the original algorithm, segment a would be in contact with both segment b and c, although there is only one contact point between the bacteria. It results in the contact twice as stiff compared to the contact where an end of the bacterium is involved. To avoid such unrealistic doubling, only one contact force is introduced in this situation. Because segments in a bacterium are ordered (i.e. the $P = 1$ end of one segment shares the same point with the $P = 0$ end of the next segment), contact forces are not introduced if a contacting segment has a contact point $P = 0$ and is in the middle of bacterium, i.e. if, for two contacting segments $\boldsymbol{Q}_{ij}$ and $\boldsymbol{Q}_{kl}$, $P_1 = 0$ and $i \neq 1$, or $P_2 = 0$ and $k \neq 1$. The only exception to this rule arises because the $P = 0$ end of the $i = 1$ segment (one of the poles of a bacterium) does not share a common point with another segment on the same bacterium, and segments $i = 1$ and $i = 2$ on the same bacterium are not checked for collision (they are adjacent and share a common point). Thus, when two contacting segments belong to the same bacterium and one of the segments is $i = 1$ with contact point $P = 0$ (a pole of a bacterium) and another segment is $i = 3$ with contact point $P = 0$ (i.e. when the first angular spring bends excessively), a collision would remain unresolved and the collision forces must be introduced.

Furthermore, in the situation depicted in S1B Fig, the end of segment a will be in contact with some non-end point of segment c, but also with the end of segment b. Contact between a and b would result not only in the desired normal contact forces on segments a and b, but also in forces in the tangential direction of the bacteria. If the bacteria moved in opposite directions, such friction forces would oppose their relative movement. This situation can be especially relevant when bacteria are under large lateral stresses that keeps them in the overlapped state. To overcome this undesirable effect, if the end of segment a makes a contact with the end of segment b, but also with some non-end point of segment c (adjacent to segment b), contact forces between a and b are not introduced. Thus, only contact forces between segments a and c that are separating the two bacteria apart would remain, the intended behavior.

Finally, in the improved algorithm, contact forces on particles $\boldsymbol{r}_i$ and $\boldsymbol{r}_{i+2}$ which were initially intended to limit an excessive bending for each angular spring in the original model [1], were removed, because in case of $N > 3$ the algorithm is capable to resolve such excessive bending of angular springs without the need of this extra assumption.

References

1. Janulevicius A, van Loosdrecht MCM, Simone A, Picioreanu C. Cell flexibility affects the alignment of model myxobacteria. Biophys J. 2010;99:3129-3138. doi:10.1016/j.bpj.2010.08.075
