# Supplementary material for: Short-Range Guiding Can Result in the Formation of Circular Aggregates in Myxobacteria Populations
Source: PLoS Comput Biol. 2015 Apr 30;11(4):e1004213. doi: 10.1371/journal.pcbi.1004213 (PMC4415783; doi:10.1371/journal.pcbi.1004213)
Supplement: S2 Text — (PDF) [file pcbi.1004213.s002.pdf]

Supporting Text S2

Short-range Guiding can Result in the Formation of Circular Aggregates in Myxobacteria Populations

Albertas Janulevicius, Mark van Loosdrecht, Cristian Picioreanu

A continuous 2D rigid body model of a circular aggregate. A circular aggregate is modeled as a rigid body with radius R rotating with constant angular speed ω. In a circular aggregate, bacteria are oriented normal to the aggregate radius and so are bacterial engine force $\boldsymbol{F}^{\mathrm{e}}$ and velocity $\boldsymbol{v}$ (S4 Fig). In the mass-spring model, the magnitude of the engine force acting on one particle is $F_{\mathrm{p}}^{\mathrm{e}} = F^{\mathrm{e}}/N$. If the area of a bacterium per particle is $a = LW/N$, then, in a continuous model, corresponding engine force on a small area element dA is $a^{-1} F_{\mathrm{p}}^{\mathrm{e}}\, dA$. Drag force $\boldsymbol{F}^{\mathrm{d}}$ acts in the opposite direction of velocity and has magnitude $a^{-1}\zeta^{\mathrm{t}} v_i^{\mathrm{t}}\, dA$.

In the head-to-tail adhesion case of guiding, adhesion forces form action-reaction pairs and are internal to the aggregate, while engine forces and drag forces acting on bacteria are the only external forces. Let us consider a circular sector of a small angle θ and its element between radii r and $r + dr$. The area of the element is

$$dA = \frac{1}{2}(r + dr)^2\theta - \frac{1}{2}r^2\theta = \frac{1}{2}\theta(2r\, dr + dr^2) \approx r\theta\, dr.$$

Torque produced by engine and drag forces on that element (assuming engine forces produce counterclockwise rotation) is

$$d\tau = (a^{-1}F_{\mathrm{p}}^{\mathrm{e}}\, dA)r - (a^{-1}\zeta^{\mathrm{t}} v_i^{\mathrm{t}}\, dA)r = a^{-1}F_{\mathrm{p}}^{\mathrm{e}} r^2\theta\, dr - a^{-1}\zeta^{\mathrm{t}}\omega r^3\theta\, dr.$$

The total torque on the circular sector is

$$\int_0^R d\tau = a^{-1}(\frac{1}{3}F_{\mathrm{p}}^{\mathrm{e}} R^3\theta - \frac{1}{4}\zeta^{\mathrm{t}}\omega R^4\theta).$$

Since the system is symmetric with respect to the rotation point and we assume the system to be in rotational equilibrium (rotating with constant ω), the total torque on the given circular sector must be zero. This results in

$$\omega R = v(R) = \frac{4F_{\mathrm{p}}^{\mathrm{e}}}{3\zeta^{\mathrm{t}}} = \frac{4}{3}v_{\mathrm{b}}.$$

In other words, the continuous model predicts that in the head-to-tail adhesion case, bacteria that are at distance R from the center of the aggregate (i.e. at the aggregate edge) should travel 4/3 their equilibrium speed v_{b}.

In case of active following, guiding forces are only added to trailing cells and therefore they do not form action-reaction pairs and are external to the system. In a circular aggregate, the component of the guiding force along the velocity vector acts to increase the speed of a bacterium and thus the angular speed of the aggregate. In the mass-spring model, the magnitude of the guiding force on a bacterium per particle can be between 0 and $F_{\mathrm{p}}^{\mathrm{g}} = F_{\mathrm{max}}^{\mathrm{g}}/N$ and depends on the distance between the tail of leading and the head of trailing bacteria. In addition, the trailing cell can interact with tails of more than one bacterium at a time. Given that each bacterium on average interacts with one tail, by adding all torques due to external forces as above, we estimate the maximum speed of cells at the aggregate edge to be

$$\omega R = v(R) = \frac{4}{3}\frac{F_{\mathrm{p}}^{\mathrm{e}} + F_{\mathrm{p}}^{\mathrm{g}}}{\zeta^{\mathrm{t}}} = \frac{4}{3}v_{\mathrm{b}}(1 + \frac{F_{\mathrm{p}}^{\mathrm{g}}}{F_{\mathrm{p}}^{\mathrm{e}}}).$$
