# Supplementary figures and images for: Short-Range Guiding Can Result in the Formation of Circular Aggregates in Myxobacteria Populations
Source: PLoS Comput Biol. 2015 Apr 30;11(4):e1004213. doi: 10.1371/journal.pcbi.1004213 (PMC4415783; doi:10.1371/journal.pcbi.1004213)

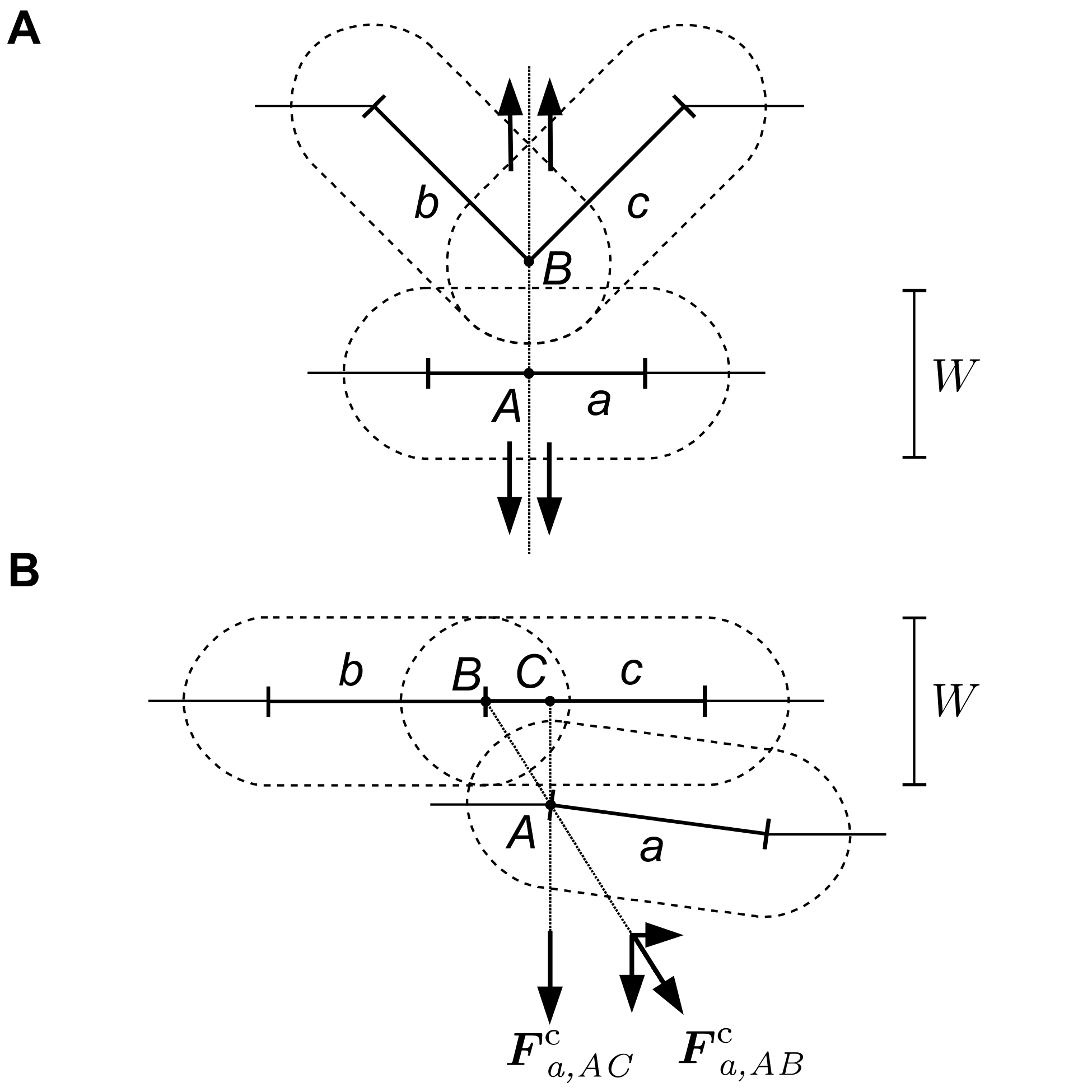

Supplement: S1 Fig — (A) Double contact forces arise because two adjacent segments share the same endpoint. (B) Friction forces arise when a segment is within contact distance with two adjacent segments on the same bacterium. AB is the shortest distance between segments a and b (i.e. A and B are contact points on the respective segments), AC is the shortest distance between segments a and c. Fa,ABc is the collision force that acts on segment a due to segment b. Fa,ACc is the collision force that acts on segment a due to segment c. (TIFF) [file pcbi.1004213.s003.tiff]

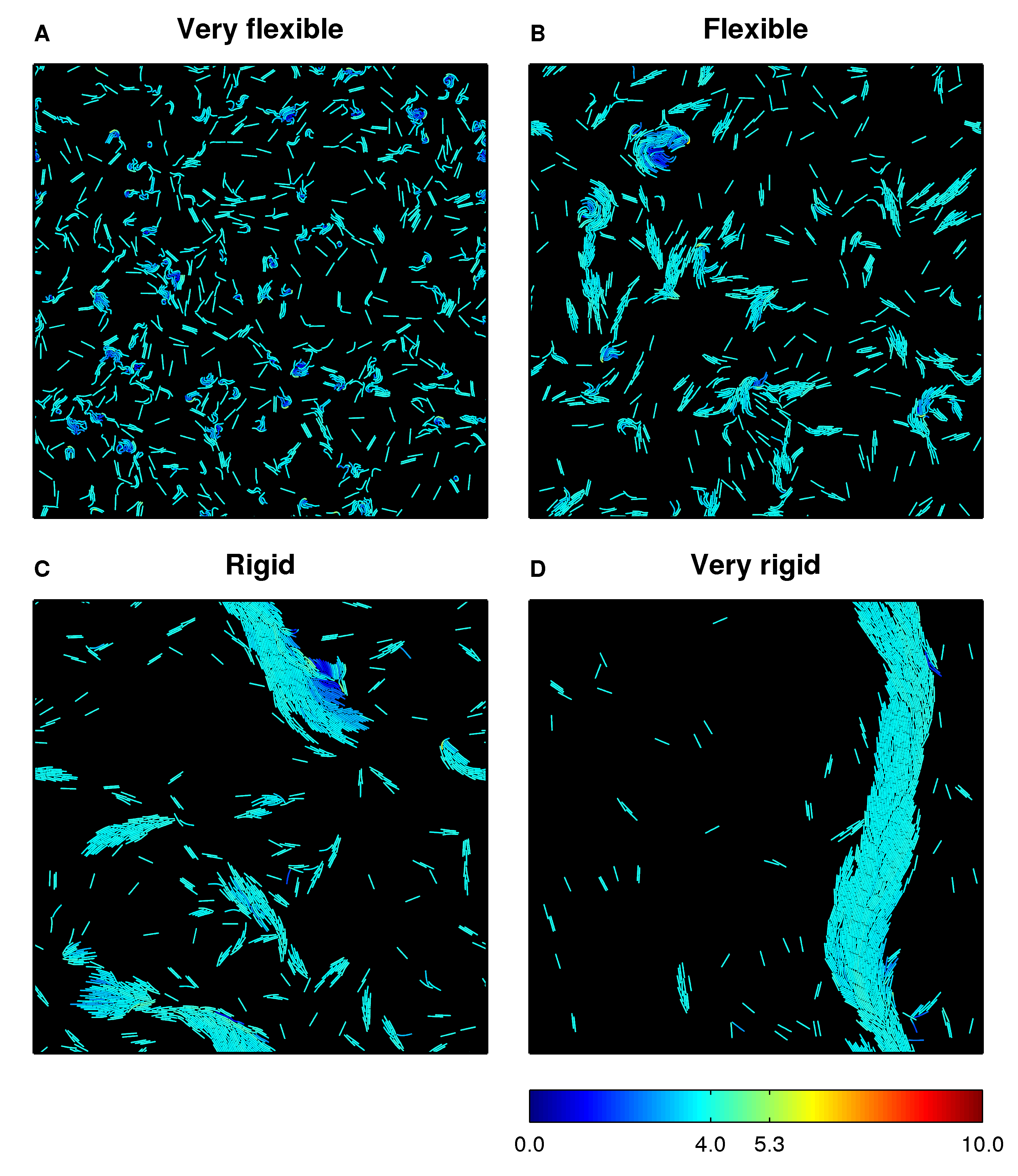

Supplement: S2 Fig — Cells were initially arranged randomly, as in Fig 3A. Final configuration of a population at 6 h is shown. Color indicates speed of individual cells (colorbar at the bottom, μm·min−1). (TIFF) [file pcbi.1004213.s004.tiff]

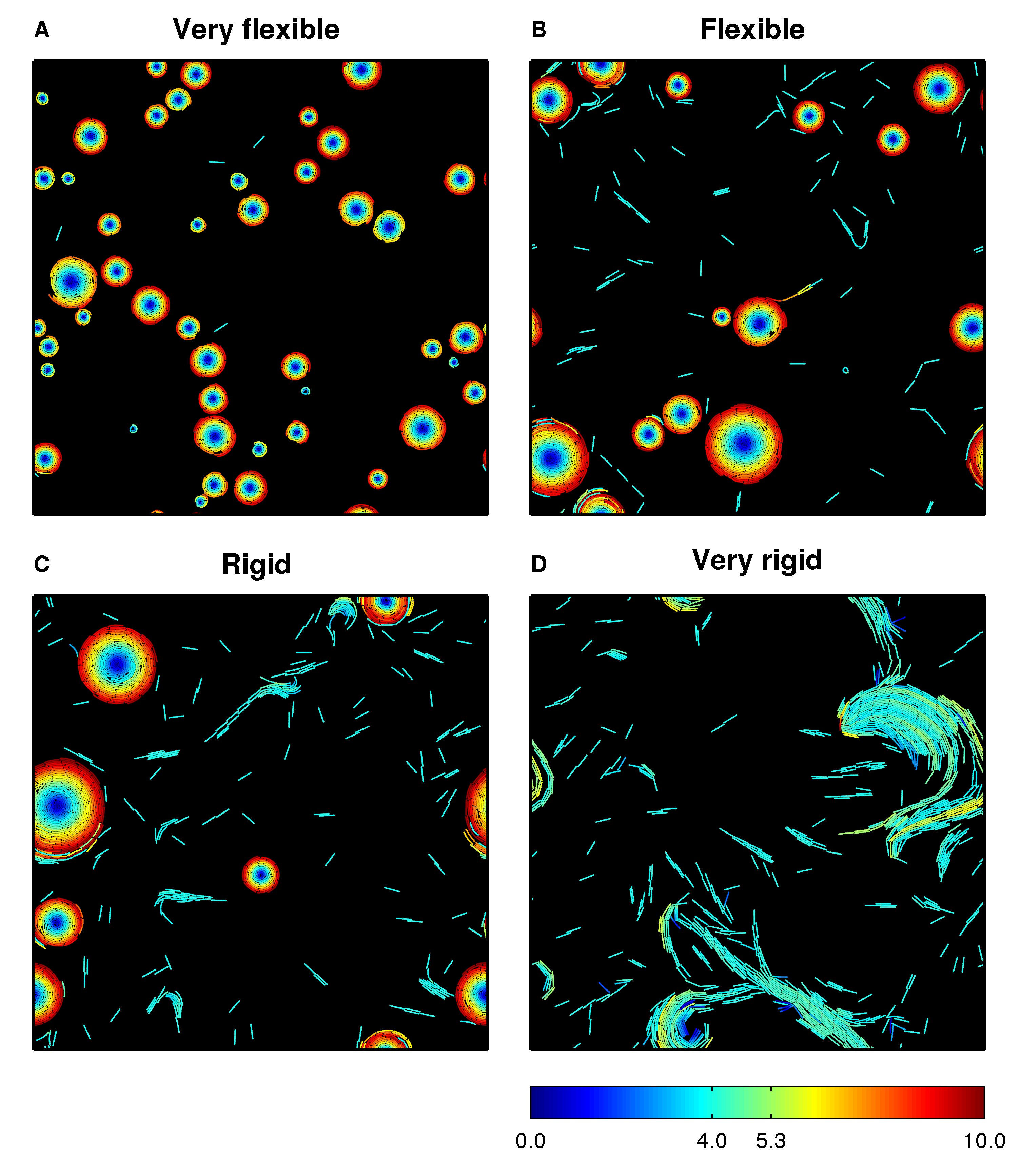

Supplement: S3 Fig — Cells were initially arranged randomly, as in Fig 3A. Final configuration of a population at 6 h is shown. Color indicates speed of individual cells (colorbar at the bottom, μm·min−1). (TIFF) [file pcbi.1004213.s005.tiff]

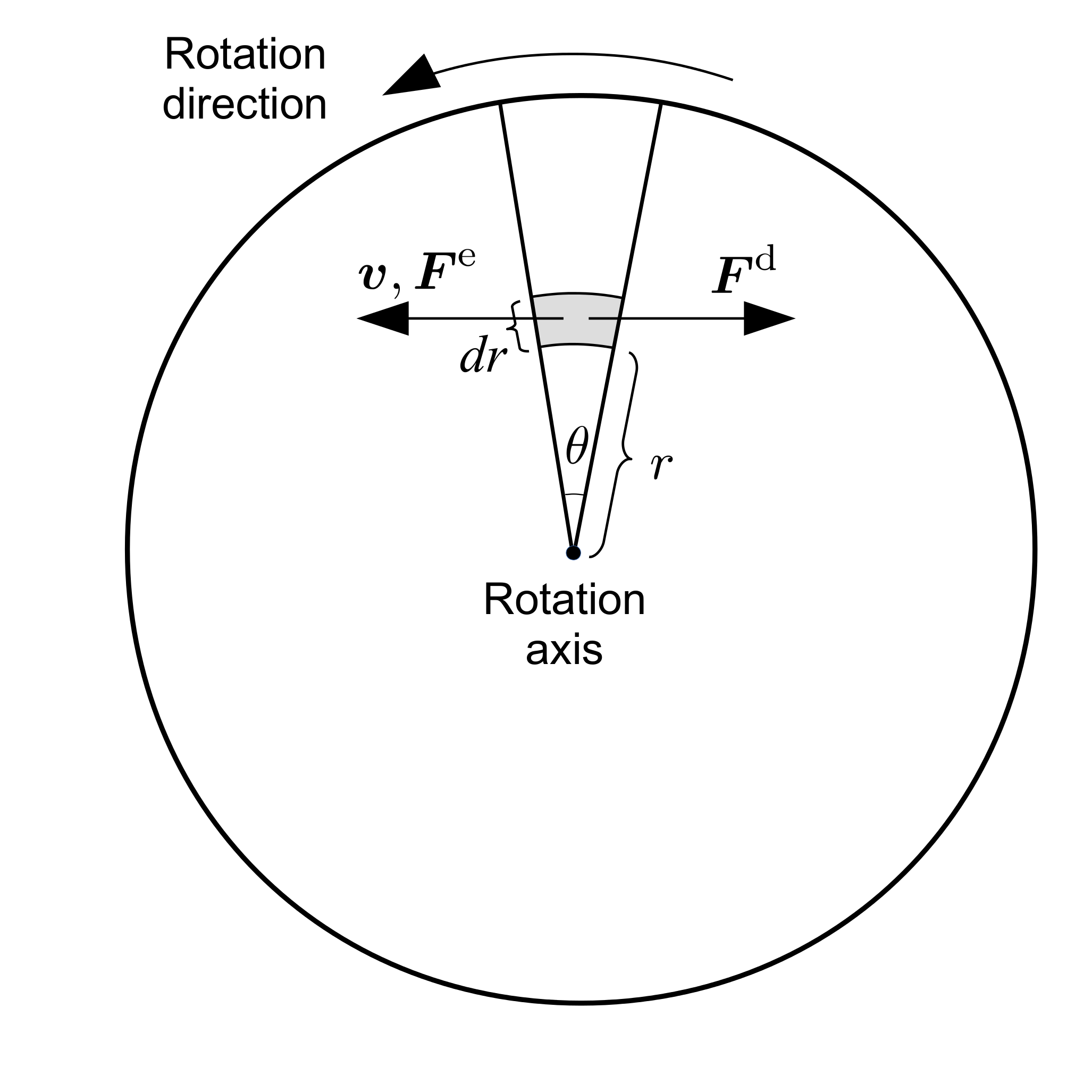

Supplement: S4 Fig — A small element of a circular sector (shaded), direction of its velocity (v), and directions of engine force (F e) and drag force (F d) that act on the element are shown. (TIFF) [file pcbi.1004213.s006.tiff]

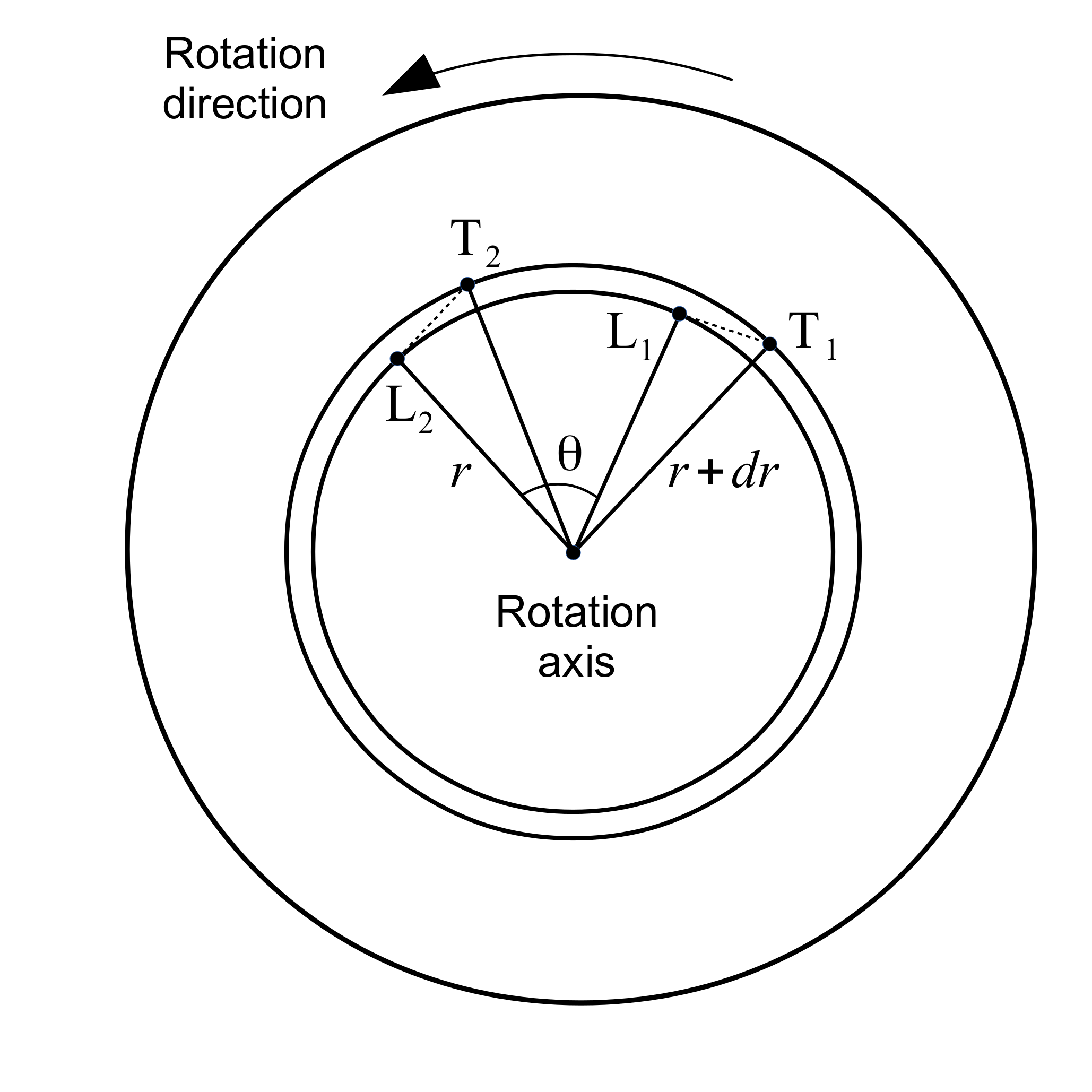

Supplement: S5 Fig — For simplicity, cells are represented as points. In a spiral arrangement of cells, each trailing cell (T) is located slightly further from the rotation axis than the respective leading cell (L). Each cell moves circularly around the rotation axis. The leading and the trailing cells must maintain a small separation for short-range guiding forces to work. Therefore, between time points 1 and 2, when the leading cell travels the distance rθ, the trailing cell must travel a longer distance, (r + dr)θ. (TIFF) [file pcbi.1004213.s007.tiff]

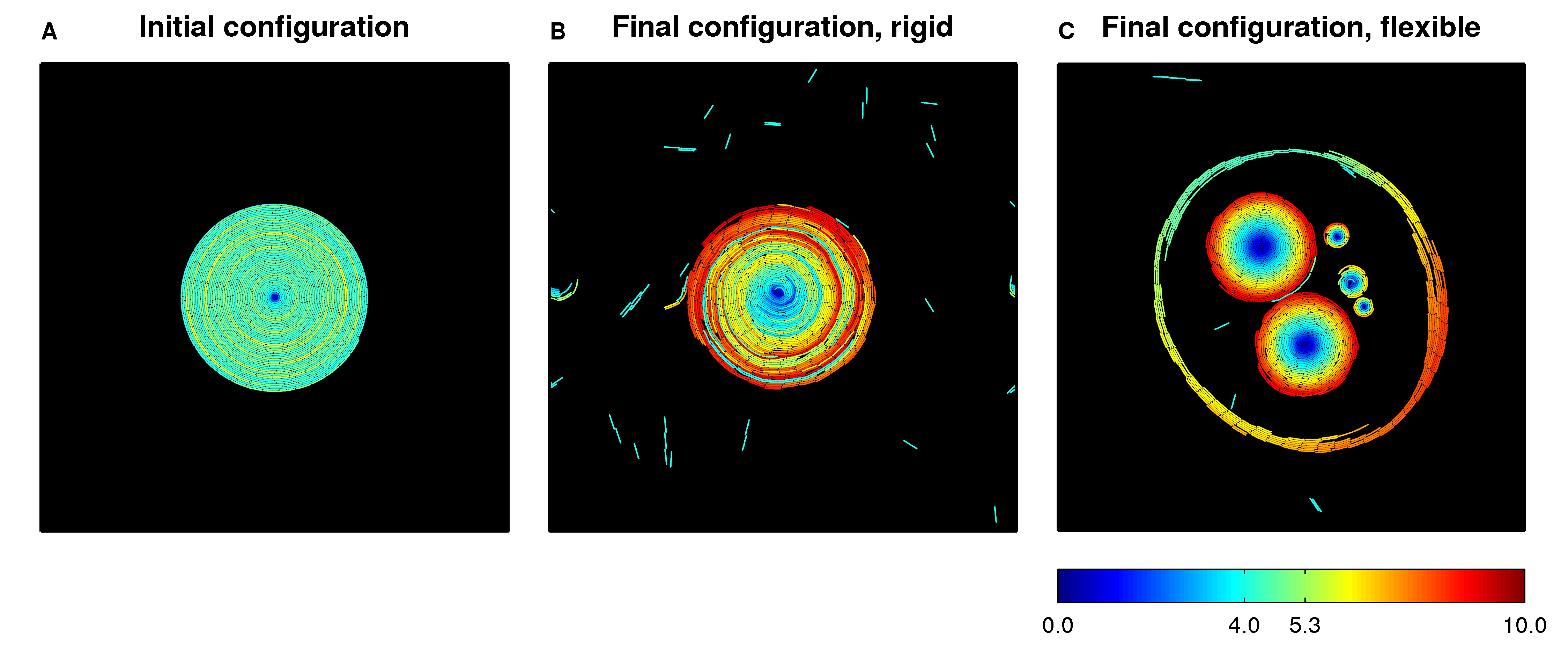

Supplement: S6 Fig — (A) Initial cell configuration. (B) Final configuration of rigid cells at 6 h. (C) Final configuration of flexible cells at 6 h. Color indicates speed of individual cells (colorbar at the bottom, μm·min−1). (TIFF) [file pcbi.1004213.s008.tiff]
